# Epigenetic silencing and deletion of the *BRCA1 *gene in sporadic breast cancer

**DOI:** 10.1186/bcr1522

**Published:** 2006-07-17

**Authors:** Valgerdur Birgisdottir, Olafur A Stefansson, Sigridur K Bodvarsdottir, Holmfridur Hilmarsdottir, Jon G Jonasson, Jorunn E Eyfjord

**Affiliations:** 1 The Icelandic Cancer Society, Molecular and Cell Biology Research Laboratory, Reykjavik, Iceland; 2 University of Iceland Department of Medicine, Reykjavik, Iceland; 3 The Icelandic Cancer Registry, Reykjavik, Iceland; 4 University Hospital Department of Pathology, Reykjavik, Iceland

## Abstract

**Introduction:**

*BRCA1 *or *BRCA2 *germline mutations increase the risk of developing breast cancer. Tumour cells from germline mutation carriers have frequently lost the wild-type allele. This is predicted to result in genomic instability where cell survival depends upon dysfunctional checkpoint mechanisms. Tumorigenic potential could then be acquired through further genomic alterations. Surprisingly, somatic *BRCA *mutations are not found in sporadic breast tumours. *BRCA1 *methylation has been shown to occur in sporadic breast tumours and to be associated with reduced gene expression. We examined the frequency of *BRCA1 *methylation in 143 primary sporadic breast tumours along with *BRCA1 *copy number alterations and tumour phenotype.

**Methods:**

Primary sporadic breast tumours were analysed for *BRCA1α *promoter methylation by methylation specific PCR and for allelic imbalance (AI) at *BRCA1 *and *BRCA2 *loci by microsatellite analysis and *TP53 *(also known as p53) mutations by constant denaturing gel electrophoresis. The *BRCA1 *methylated tumours were analysed for *BRCA1 *copy alterations by fluorescence *in situ *hybridisation and BRCA1 expression by immunostaining.

**Results:**

*BRCA1 *methylation was found in 13/143 (9.1%) sporadic breast tumours. The *BRCA1 *methylated tumours were significantly associated with estrogen receptor (ER) negativity (P = 0.0475) and displayed a trend for *BRCA1 *AI (P = 0.0731) as well as young-age at diagnosis (≤ 55; P = 0.0898). *BRCA1 *methylation was not associated with *BRCA2 *AI (P = 0.5420), although a significant association was found between *BRCA1 *AI and *BRCA2 *AI (P < 0.0001).

Absent/markedly reduced BRCA1 expression was observed in 9/13 *BRCA1 *methylated tumours, most of which had *BRCA1 *deletion. An elevated *TP53 *mutation frequency was found among *BRCA1 *methylated tumours (38.5%) compared with non-methylated tumours (17.2%). The *BRCA1 *methylated tumours were mainly of tumour grade 3 (7/13) and infiltrating ductal type (12/13). Only one methylated tumour was of grade 1.

**Conclusion:**

*BRCA1 *methylation is frequent in primary sporadic breast tumours. We found an indication for *BRCA1 *methylation to be associated with AI at the *BRCA1 *locus. Almost all *BRCA1 *methylated tumours with absent/markedly reduced BRCA1 expression (8/9) displayed *BRCA1 *deletion. Thus, epigenetic silencing and deletion of the *BRCA1 *gene might serve as Knudson's two 'hits' in sporadic breast tumorigenesis. We observed phenotypic similarities between *BRCA1 *methylated and familial *BRCA1 *tumours, based on *BRCA1 *deletion, *TP53 *mutations, ER status, young age at diagnosis and tumour grade.

## Introduction

Germline mutations in one allele of the *BRCA1 *or *BRCA2 *genes significantly increase the risk of developing early-onset breast cancer [1]. Tumour cells from predisposed individuals have consistently lost the wild-type *BRCA *allele [2,3]. The most prominent feature of BRCA deficient cells is the inability to repair DNA cross-links and DNA double-strand breaks by error-free homologous recombination, which probably underlies genomic instability and cancer predisposition [4]. Survival of BRCA deficient cells is generally thought to be dependent upon dysfunctional checkpoint mechanisms, in which case tumorigenic potential could be acquired through additional genomic rearrangements and gene mutations. Indeed, familial *BRCA1 *tumours are associated with mutations in the *TP53 *checkpoint gene, supporting the notion that genomic instability is an important driving force in early-onset familial *BRCA1 *tumorigenesis [5].

Although inherited cancer syndromes are rare, the genes accounting for them are generally believed to play an important role in sporadic cancer. It was anticipated, therefore, that somatic *BRCA *mutations would be found to contribute to sporadic breast carcinogenesis. Surprisingly, somatic *BRCA *gene mutations have not been found in sporadic breast tumours [6,7]. On the other hand, allelic imbalance (AI) at the *BRCA *loci, an indicator for loss of heterozygosity, is know to be a fairly common event in breast cancer [8]. The implications of AI at the *BRCA *loci are unknown since Knudson's hypothesis predicts an additional inactivating event on top of AI to be required for tumorigenesis to occur [9]. For these reasons, the involvement of the *BRCA *genes in sporadic breast tumours has been questioned. An alternative mechanism for *BRCA1 *inactivation has been suggested to be gene silencing by epigenetic mechanisms. Hypermethylation of CpG-island promoters is known to be strongly associated with gene silencing. Once established, methylation is passed on to daughter cells during DNA replication by the activity of DNA methyltransferases, thereby conserving the overall pattern of methylated CpG-islands [10]. The methylation patterns of virtually all types of cancer, including breast carcinoma, have been found to differ extensively from that of the corresponding normal tissue. These alterations are cancer-type specific and include global genomic hypomethylation as well as non-random hypermethylation of normally unmethylated CpG-island promoters [11,12]. These observations, and others, indicate that epigenetic modifications could be important in cancer etiology [13].

Several studies have reported hypermethylation of the *BRCA1 *promoter in sporadic breast and ovarian tumours. Furthermore, *BRCA1 *methylation has only been found in breast and ovarian tumours and has been associated with AI at the *BRCA1 *locus and reduced *BRCA1 *gene expression [12,14]. *BRCA2 *promoter hypermethylation has not been found in breast tumours, although it has been reported in ovarian tumours [15,16].

Familial *BRCA1 *and *BRCA2 *tumours are associated with young age of onset and are phenotypically distinct from each other as well as from sporadic breast tumours [1,17-19]. Conventional histopathological and molecular analyses have demonstrated familial *BRCA1 *tumours to have a basal-like phenotype and to be significantly associated with certain features, such as AI at the *BRCA1 *locus, a negative estrogen receptor (ER) and progesterone receptor (PR) status, a medullary tumour histological type, *TP53 *mutations and, depending on the mutation involved, a high tumour grade [3,5,18,20,21]. Interestingly, gene expression profiling has revealed similarities between *BRCA1 *methylated and familial *BRCA1 *tumours [17,22]. Similarly, a comparative genomic hybridisation study has reported a specific pattern of genetic alterations to be predictive of familial *BRCA1 *tumours and *BRCA1 *methylated tumours [23]. This lends support to the idea that epigenetic silencing of the *BRCA1 *gene might channel tumour progression, akin to an underlying *BRCA1 *germline mutation resulting in a *BRCA*-like phenotype. However, a recent report showing high levels of BRCA1 expression and a low frequency of *BRCA1 *promoter methylation in basal-like sporadic tumours suggests that this might be more complex [24].

In the present study, we examined the frequency of *BRCA1*α promoter hypermethylation in 143 unselected primary sporadic breast tumours. All tumours were analysed for AI at the *BRCA1 *and *BRCA2 *loci, *TP53 *mutations, hormonal receptor status and age at diagnosis. Copy number alterations at the *BRCA1 *locus were further examined by fluorescence *in situ *hybridisation (FISH) in the *BRCA1 *methylated tumours which were also analysed for BRCA1 protein expression, histological type and tumour grade. The purpose of the study was to examine whether the *BRCA1 *gene could be implicated in sporadic breast tumorigenesis through epigenetic modifications.

## Materials and methods

### Study group

The study group consisted of 143 female breast cancer patients that carried neither the Icelandic *BRCA1 *5193G→A nor the *BRCA2 *999del5 germline mutations [25,26]. DNA samples from these patients were obtained from the Biological Specimen Bank of the Icelandic Cancer Society. Tumour DNA (obtained from fresh/frozen primary breast cancer tissue) and normal DNA (obtained from blood or from fresh/frozen breast tissue adjacent to the breast cancer tissue) were available from each of the patients. Data on tumour grade (Nottingham tumour grade), histological type, ER and PR status, flow-cytometric DNA index and aneuploidy of the tumours were obtained from the Department of Pathology, Landspitali University Hospital (Reykjavik, Iceland). This work was carried out according to permits from the Icelandic Data Protection Commission (2004040264; 200403147) and Bioethics Committee (99041V2S1; 99111V1S1).

### Methylation specific PCR

DNA methylation of the *BRCA1 *promoter region was assessed by methylation specific PCR of sodium bisulphite treated DNA [27]. Tumour DNA and controls (1 μg) were treated with sodium bisulphite and purified using the Wizard DNA Clean-Up System (catalogue no. A7280, Promega, Madison, WI) following the manufacturer's recommendations. Modified DNA was amplified with published PCR primers that distinguish unmethylated and methylated DNA. Primer sequences for unmethylated and methylated DNA were as follows: unmethylated forward, ggt taa ttt aga gtt ttg aga gat g; unmethylated reverse, t caa caa act cac acc aca caa tca; methylated forward, ggt taa ttt aga gtt tcg aga gac g; and methylated reverse, tca acg aac tca cgc cgc gca atc g [28]. The primers amplified a 182 base-pair (bp) product corresponding to nucleotides -150 to +32 relative to the main transcription start site of *BRCA1*. DNA extracted from blood was used as a negative control for methylated *BRCA1 *alleles. DNA extracted from blood and methylated *in vitro *was used as a positive control. The PCR solution (15 μl) contained 1 μl of modified DNA in 1X Thermo-Start PCR Master Mix (ABgene, Epsom, UK) and 5 pmol of each primer. The PCR was carried out in a thermocycler with the following conditions: one cycle of 95°C for 15 minutes followed by 35 cycles of 94°C for 30s, 65°C for 30s and 72°C for 60s, ending with one cycle of 72°C for 5 minutes. Then, 6 μl of the PCR product were mixed with 6 μl of 1X loading buffer (98% formamide, 0.1% xylene cyanol, 0.1% bromophenol blue and 10 mM EDTA) and electrophorised on 7.5% polyacrylamide gels.

### Allelic imbalance by microsatellite analysis

AI at polymorphic microsatellite markers was analysed by laser quantification of PCR products. We analysed two intragenic *BRCA1 *markers (D17S855 and D17S1323), located within introns 12 and 20, respectively, and one marker centromeric to the *BRCA1 *gene (D17S846) located in region 17q12. Two *BRCA2 *markers were analysed, located in region 13q12, centromeric (D13S260) and telomeric (D13S171) to the gene. The marker primers were of published sequences available from The GDB Human Genome Data Base [29]. The primers were purchased HPLC purified from Eurogentec (Seraing, Belgium) with the forward primers Cy5 indocarbocyanin labelled.

The PCR solution (15 μl) contained 50 ng of DNA, 5 pmol of each primer, 0.2 mM Ultrapure dNTPs (Amersham Pharmacia, Little Chalfont, Buckinghamshire, UK) and 0.36U Dynazyme enzyme mix (Finnzymes, Espoo, Finland) with supplied 1X reaction buffer. A Hot Start was performed by heating the PCR solution in a thermocycler at 94°C for 2 minutes and cooling to 85°C before the enzyme was added to the solution. This was followed by 30 cycles at 94°C for 30s, 64°C to 69°C (annealing temperature varied depending on which primers were used) for 30s and 72°C for 60s, ending with 1 cycle of 72°C for 5 minutes.

The PCR products were mixed in a stop solution (100% deionized formamide and Dextran Blue 2000 (5 mg/ml); Amersham Pharmacia) in ratios varying from 0.13 to 1, denatured at 95°C for 5 minutes and resolved on a 3 mm thick High Resolution Reprogel (Amersham Pharmacia) using an automated laser fluorescent sequencer (ALF Express DNA Sequencer, Amersham Pharmacia). Aliquots of 3 to 5 μl of each sample were loaded onto the gel. The following running parameters were used: 1,500 V, 60 mA, 25 W, 55°C. The sample interval was 2s, the running time 300 minutes and the running buffer 1X TBE (Tris-BoricAcid-EDTA). ALFwin Fragment analyser 1.0 software (Amersham Parmacia) was used to compare the relative quantity of the PCR products. AI was defined if the relative difference of peaks representing alleles in the tumour and the corresponding normal DNA reactions was more than 25%.

### Fluorescence *in situ *hybridization

FISH analysis was performed on paraffin embedded and formalin fixed breast tumour tissue sections (sliced in 4 μm sections) using DNA probes for the *BRCA1 *region and the centromere region of chromosome 17, simultaneously. The probe for the *BRCA1 *region (PAC103014; the Human BAC Clone Library, Sanger Centre, Hinxton, Cambridge, UK) which spans the entire *BRCA1 *gene was labelled with SpectrumOrange-dUTP (Vysis, Des Plaines, IL, USA) by nick-translation. Hybridisation efficiency of the *BRCA1 *probe has previously been tested in non-malignant breast samples [30]. The probe for the centromere region of chromosome 17 (clone D17Z1 in pUC 19: American Type Culture Collection, USA), was used as a copy number reference for *BRCA1 *and labelled with green fluoroscein-11-dUTP (Amersham Pharmacia) by nick-translation.

Tissue sections were deparaffinized, placed in 0.01 M citric acid solution (pH 6) and heated for 2 × 10 minutes in a microwave oven at maximum power. After cooling, tissue sections were incubated with pepsin at 37°C for 20 minutes followed by dehydration. Probes were diluted in t-DenHyb-2 hybridisation buffer (InSitus Biotechnologies, Albuquerque, NM, USA) as described by the manufacturer. Tissue section chromosomes and probes were simultaneously denatured at 95°C for 10 minutes. This was followed by overnight hybridisation at 37°C in a humid chamber and washing of tissue sections for 3 × 5 min in 0.1X SSC (Saline-Sodium-Citrate) at 60°C and mounting with 4'-6-Diamidino-2-phenylindole (DAPI) counterstaining. Fluorescence signals were scored in each sample by counting the number of single-copy gene and centromeric signals in at least 100 well-defined nuclei. Deletion of *BRCA1 *was defined if the copy number ratio was 0.8 or less, which has previously been used to detect deletion [30]. Deletion of chromosome 17 was defined if both *BRCA1 *and centromere mean copy numbers were 1.5 or less.

### Immunohistochemistry

BRCA1 protein expression analysis was performed on formalin fixed and paraffin embedded malignant breast tissue and adjacent normal tissue (sliced in 4 μm sections), with BRCA1 MS110 antibody (Oncogene Research Products, San Diego, CA, USA). Tissue sections were deparaffinized, placed in 0.01 M citric acid solution (pH 6) and heated for 2 × 10 minutes in a microwave oven at maximum power. The sections were then incubated in 3% H_2_O_2 _in order to block endogenous peroxidase activity. The BRCA1 MS110 antibody (100 μg/ml) was used in 1:50 dilution in 1X Tris buffer and incubated in a humid chamber at room temperature overnight. For antibody detection all slides were incubated with StreptABComplex/HRP Duet, Mouse/Rabbit Kit (Code No. K0492: Dako, Glostrup, Denmark) reagents following the manufacturer's recommendations. Counterstaining was performed with haemotoxylin.

Positive staining of normal breast epithelial cells that either co-existed on the tumour sections and/or normal breast tissue sections from the same breast was used as a control. The protein expression levels in tumour sections were measured by eye in three discontinuous classes, as previously described [31]. When the immunoreactivity was comparable to that of the normal breast epithelium or nuclear staining was observed in >50% of tumour cells, it was classified as level 3, that is, wild-type expression. When the staining was clearly weaker than normal surrounding cells or nuclear staining occurred in 20% to 50% of tumour cells, it was classified as level 2, that is, reduced expression. When there was no staining or nuclear staining occurred in <20% of tumour cells, it was classified as level 1, that is, absent/markedly reduced expression.

### *TP53 *mutation analysis

*TP53 *mutation analysis was carried out by PCR amplification and constant denaturing gel electrophoresis on exons 5–8. Mutations were confirmed by direct DNA sequencing in an ALF Express DNA Sequencer (Amersham Pharmacia) as previously described [32].

### Statistical analysis

Proportions were compared by two-tailed Fisher's exact test using GraphPad InStat3 (GraphPad Software Inc., San Diego, CA, USA). Associations with P values of <0.05 were considered to be significant and P values within the range of 0.05 to 0.10 as an indication of an association.

## Results

### Methylation of the *BRCA1 *promoter

**Figure 1:**
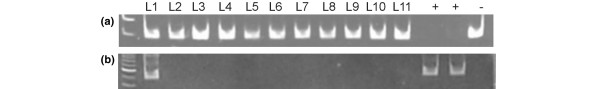
Methylation-specific PCR analysis of the *BRCA1 *promoter region. Positive (+) and negative (-) controls are seen on the far right. Each lane contains products generated from separate PCR reactions using primers specific for **(a) **non-methylated and **(b) **methylated *BRCA1 *alleles. The tumour sample in lane 1 (L1) shows the presence of a band in both PCR reactions, indicating methylation of the *BRCA1 *promoter region.

Hypermethylation of the *BRCA1*α promoter was assayed in 143 primary sporadic breast tumours. Methylation was detected in 9.1% (13/143) of the tumours (Figure 1).

### Allelic imbalance at the *BRCA1 *and *BRCA2 *loci

**Figure 2:**
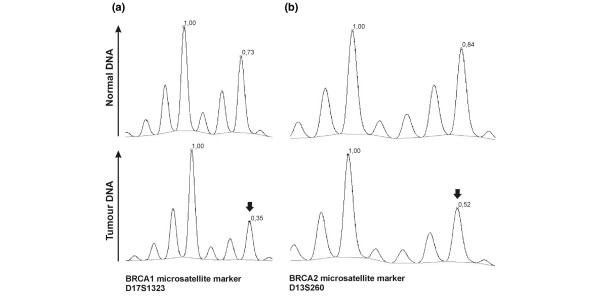
Allelic imbalance analysis at the *BRCA *loci. The electropherograms represent quantification of PCR products from normal DNA (upper) and tumour DNA (lower) from the same individual. A signal reduction is evident in the tumour electropherograms, which suggest allelic imbalance (indicated by an arrow) at the **(a) ***BRCA1 *locus, using the D17S1323 marker, and **(b) ***BRCA2 *locus, using the D13S260 marker.

AI in the *BRCA1 *region was assessed in all 143 samples (Figure 2a,b). The frequencies of informative cases for polymorphism at the *BRCA1 *microsatellite marker regions were 72.7% (D17S846), 89.5% (D17S855) and 40.6% (D17S1323). The intragenic markers D17S855 and D17S1323 showed AI in 35.2% and 43.1% of informative cases, respectively (Additional file 1). The exogenic marker D17S846 showed AI in 36.5% of informative cases. AI at the *BRCA1 *locus was defined if one or both of the intragenic markers (D17S855, D17S1323) displayed AI. According to this definition, 37.4% (49/131) of informative tumours had AI at the *BRCA1 *locus. Of these 49 tumours, 24 were informative for both intragenic markers, of which all but one displayed AI at both regions (95.8%). Of the cases informative for an intragenic marker and the exogenic marker D17S846 (*n *= 33), 87.9% displayed AI at both regions.

An indication was found for an association between *BRCA1 *methylation and AI at the *BRCA1 *locus (P = 0.0731, odds ratio (OR) = 3.0, 95% confidenece interval (CI) = 0.9–9.8; Table 1). Of the eight *BRCA1 *methylated tumours that displayed AI at the *BRCA1 *locus, five were informative for an intragenic marker and the exogenic marker. All these five tumours displayed AI at both regions.

The frequencies of informative cases for polymorphism at the *BRCA2 *microsatellite marker regions D13S260 and D13S171 were 74.1% and 71.3%, respectively. The D13S260 marker showed AI in 32.1% of informative cases, and the D13S171 marker in 35.3% of informative cases (Additional file 1). AI at the *BRCA2 *locus was defined if one or both markers displayed AI. According to this definition, 31.1% (42/135) of informative tumours had AI at the *BRCA2 *locus. Of these 42 tumours, 29 were informative for both markers, of which all but one displayed AI at both regions (96.6%).

AI at the *BRCA1 *locus was found to be strongly associated with AI at the *BRCA2 *locus (P < 0.0001, OR = 7.0, 95%CI = 3.0–16.4) with 26 of 124 (21.0%) informative tumours having AI at both loci (Table 1). However, AI at the *BRCA2 *locus was not found to be associated with *BRCA1 *methylation (P = 0.5420, OR = 1.4, 95%CI = 0.3–5.3).

### FISH analysis at the *BRCA1 *locus

**Figure 3:**
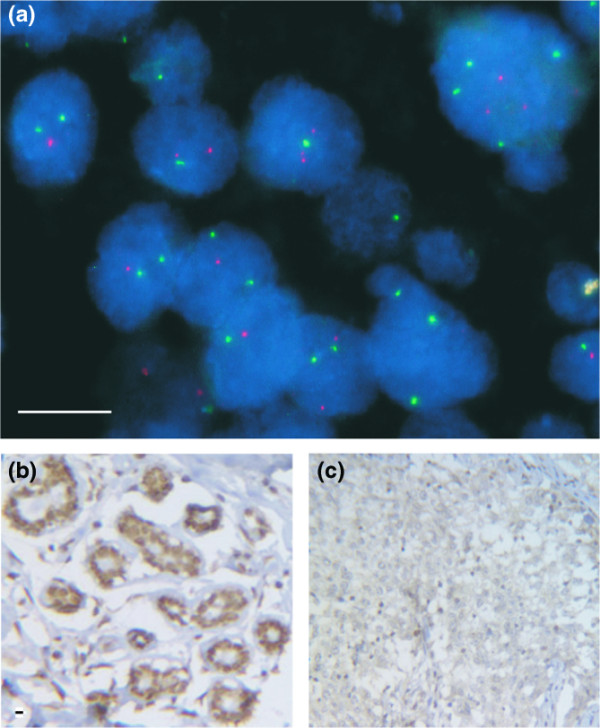
*BRCA1 *gene copy number and protein expression analysis on breast tumor sections. **(a) **Tumor cells in a *BRCA1 *methylated sample are seen to have reduced *BRCA1 *gene copy numbers (red signal) compared with centromere 17 (green signal) by FISH. Considerable heterogeneity in *BRCA1 *gene copy numbers was evident in all tumours analysed. **(b) **A *BRCA1 *methylated tumour section showing wild-type BRCA1 protein expression by immunostaining in normal breast ducts and **(c) **absent/markedly reduced BRCA1 protein expression in tumour cells. Bars = 7 μm.

*BRCA1 *gene copy number was determined in the *BRCA1 *methylated tumours by FISH analysis (Additional file 1). Considerable heterogeneity was evident in the nuclei of these tumour cells (Figure 3a).

A physical deletion was detected at the *BRCA1 *locus in six tumours, including four with deletion of chromosome 17 (Table 2). Of these six tumours, all but one (sample 6 in Table 2) showed AI at the *BRCA1 *locus.

### BRCA1 protein expression

BRCA1 protein expression was estimated in all *BRCA1 *methylated tumours by immunostaining. Nine of the methylated tumours were estimated to have class 1 BRCA1 protein expression, indicating absent or markedly reduced BRCA1 expression (Figure 3b,c; Table 2). Of these nine tumours, all but two had AI at the *BRCA1 *locus (Table 2). Four tumours were estimated to have class 2 or 3 BRCA1 protein expression (Table 2). Of these four tumours, AI at the *BRCA1 *locus was detected in one case (Table 2).

### *TP53 *mutation analysis

Of the 143 primary sporadic breast tumours in this study, 141 were available for *TP53 *mutation analysis. Mutation was found in 19.1% (27/141) of the tumours. Although not statistically significant, we found the frequency of *TP53 *mutations to be much higher within the subset of *BRCA1 *methylated tumours compared with the non-methylated *BRCA1 *tumours or 38.5% (5/13) compared to 17.2% (22/128), respectively (P = 0.1299, OR = 3.0, 95%CI = 0.9–10.1; Table 1). However, *TP53 *mutations were only found in those *BRCA1 *methylated tumours that exhibited absent or markedly reduced BRCA1 expression, in which case the *TP53 *mutation frequency becomes 55.5% (5 of 9) and the association statistically significant (P = 0.01317, OR = 6.13, 95%CI = 1.21–33.51; Table 2). All the five tumours with *BRCA1 *methylation and *TP53 *mutation were found to have AI at the *BRCA1 *locus (Table 2). Furthermore, all five tumours showed absent or markedly reduced BRCA1 protein expression (Table 2).

### Hormonal receptor status and age at diagnosis

Association was found between a negative ER status and *BRCA1 *methylation (P = 0.0475, OR = 3.4, 95%CI = 1.1–10.9; Table 1). No associations were found between *BRCA1 *methylation and a negative/positive PR status.

An indication for an association between young age (= 55) at diagnosis and *BRCA1 *methylation was found (P = 0.0898, OR = 2.9, 95%CI = 0.8–13.4).

### Tumour grade and histological type

Of all the 13 *BRCA1 *methylated tumours, seven were of grade 3, five of grade 2 and one of grade 1. All *BRCA1 *methylated tumours were of infiltrating ductal type except for one that was of a lobular type.

## Discussion

We report here that hypermethylation of the *BRCA1 *gene promoter is found in a considerable proportion of primary sporadic breast carcinomas, that is, 13 of 143 (9.1%), which is in the lower end of previously reported frequencies for this alteration in sporadic breast tumours [14,33,34].

Absent or markedly reduced BRCA1 protein expression was evident in the majority of the *BRCA1 *methylated tumours (9 of 13), suggesting transcriptional silencing in these tumours by epigenetic modifications. A trend for AI at the *BRCA1 *locus was observed in the subset of *BRCA1 *methylated tumours (P = 0.0731). All the *BRCA1 *methylated tumours that had AI at the *BRCA1 *locus and were informative for AI at the exogenic and an intragenic marker displayed AI at both regions, indicating a rather large deletion at chromosome 17. This is supported by the FISH analysis, which revealed deletion of chromosome 17 in most of the *BRCA1 *methylated tumours that had a detectable *BRCA1 *deletion. Importantly, the FISH analysis revealed substantial heterogeneity in *BRCA1 *gene copy numbers between individual cells in the *BRCA1 *methylated tumours, demonstrating that AI as detected by polymorphic microsatellite PCR analysis does not infer a simple loss of one *BRCA1 *allele but, rather, it appears to reflect complex genetic rearrangements.

AI at the *BRCA1 *and *BRCA2 *loci are know to be relatively common in breast tumours [8]. The implications of AI at the *BRCA1 *and/or *BRCA2 *loci for sporadic breast tumorigenesis remain unknown since Knudson's hypothesis predicts that two 'hits' are required for tumorigenesis to occur [9]. Our results confirm that AI at the *BRCA1 *and *BRCA2 *loci are common events in sporadic breast tumours, present in 37.4% (49/131) and 31.1% (42/135) of primary sporadic breast tumours, respectively. A significant association was found between AI at the *BRCA1 *and *BRCA2 *loci (P < 0.0001). Importantly, we found an indication for AI at the *BRCA1 *locus to be associated with *BRCA1 *methylation (P = 0.0731) whereas AI at the *BRCA2 *locus was not found to be associated with *BRCA1 *methylation (P = 0.5420). This has not been shown previously and suggests that AI at the *BRCA1 *locus is specifically associated with *BRCA1 *methylation. Thus, copy number alterations and epigenetic silencing of the *BRCA1 *gene in sporadic breast cancer could serve as Knudson's 'hits', which has previously been proposed by Esteller and colleagues [35]. Indeed, all but one of the *BRCA1 *methylated tumours that had absent/markedly reduced BRCA1 protein expression (8 of 9) also had a detectable deletion of the *BRCA1 *gene. Collectively, these results suggest that the *BRCA1 *gene is implicated in sporadic breast tumorigenesis through epigenetic silencing and deletion of the *BRCA1 *gene. Indications that *BRCA1 *methylation is important in hereditary breast cancer have been reported [35].

The failure to detect a *BRCA1 *deletion in one of the tumours that exhibited absent or markedly reduced BRCA1 expression could mean that promoter hypermethylation is present on both alleles, thereby alleviating any selection pressure for deletion at the *BRCA1 *locus. Alternatively, the level of detection in the FISH analysis could be limited by the small proportion of tumour cells present in each tumour section analysed. This might also apply for those tumours in which AI was present without a detectable deletion by FISH. Conversely, the detection level of the AI analysis was limited by the fact that none of the tumours were micro/macrodissected prior to DNA isolation, which also means that unmethylated *BRCA1 *alelles are always detected in the tumour samples due to the presence of normal DNA.

The four *BRCA1 *methylated tumours that did not exhibit significantly reduced BRCA1 expression could possibly be heterogenous with respect to this alteration. None of the four tumours exhibited *BRCA1 *deletion by FISH and only one displayed AI at the *BRCA1 *locus. Alternatively, DNA methylation might not bring about transcriptional silencing in all instances.

Although the etiology of cancer predisposition in individuals carrying a germline *BRCA1 *mutation is not clear, increased genomic instability in BRCA1 deficient cells is undoubtedly of importance since it is predicted to result in increased probability of further genetic alterations and gene mutations, which might result in functional consequences by which tumorigenic potential could be acquired. Genomic instability, however, is a potent inducer of apoptosis where cell survival is dependent upon dysfunctional checkpoint mechanisms [4]. Indeed, familial *BRCA1 *tumours are associated with mutations in the *TP53 *checkpoint gene, supporting the notion that genomic instability is an important driving force in early-onset familial *BRCA1 *tumorigenesis [5]. Association of *BRCA1 *methylation with *TP53 *mutations has not been shown previously. Our results show a higher frequency of *TP53 *mutations among the *BRCA1 *methylated tumours compared with the non-methylated tumours or 38.5% (5 of 13) and 17.2% (22 of 128), respectively (P = 0.1299, OR = 3.0, 95%CI = 0.9–10.1). This association was not statistically significant, although the *TP53 *mutations were found to be entirely limited to those *BRCA1 *methylated tumours that exhibited absent or markedly reduced BRCA1 expression, in which case the frequency of *TP53 *mutations becomes 55.5% (5 of 9) and the association statistically significant (P = 0.01317, OR = 6.13, 95%CI = 1.21–33.51). Reinforcing this idea is the observation that all the five *BRCA1 *methylated tumours with a *TP53 *mutation had a detectable *BRCA1 *copy number reduction and the majority of these tumours had a relatively high DNA index, suggesting genomic instability (Table 2).

It has previously been suggested that *BRCA1 *methylated tumours might phenocopy familial *BRCA1 *tumours [36]. In support of this notion, we observed ER negativity to be significantly associated with *BRCA1 *methylation (P = 0.0475), a well established characteristic of familial *BRCA1 *tumours previously reported by Catteau and colleagues [37] and others. However, Matros and colleagues [24], looking at gene expression profiles, found a high frequency of *BRCA1 *promoter methylation among high-grade ER positive tumours, suggesting a more complex phenotype association. We found an indication for *BRCA1 *methylation to be specifically associated with AI at the *BRCA1 *locus and an elevated frequency of *TP53 *mutations, which has not been reported previously. In addition, we found a considerable proportion of the *BRCA1 *methylated tumours (7 of 13) to be of grade 3, with only one tumour of grade 1, as well as an indication of an association between *BRCA1 *methylation and an early age of onset (P = 0.0898) as previously reported by Wei and colleagues [34].

It has been suggested that breast cancers arising in individuals carrying a germline mutation in the *BRCA *genes could benefit from therapeutic agents that lead to DNA cross-links or double-strand breaks at replication forks, for example, mitomycin C, cisplatin, diepoxybutane and, more recently, poly(ADP-ribose) polymerase (PARP) inhibitors [38]. These therapeutic agents could also be effective for sporadic breast cancers with abnormalities in the *BRCA *genes, which is, as shown here, a considerably larger proportion of all breast cancer patients than germline *BRCA1 *or *BRCA2 *mutation carriers. In addition, abnormalities in other genes regulating homologous recombination could also be of relevance. This emphasizes the importance of developing methods for identifying BRCA-like cancers, regardless of the underlying alterations [36].

## Conclusion

Our results show promoter hypermethylation of the *BRCA1 *gene in a considerable proportion of all primary sporadic breast tumours. The majority of the *BRCA1 *methylated tumours were found to have absent or markedly reduced BRCA1 expression, suggesting transcriptional silencing by epigenetic modifications. In addition, we found an indication for AI at the *BRCA1 *locus to be associated with *BRCA1 *methylation whereas AI at the *BRCA2 *locus was not associated with *BRCA1 *methylation. This indicates that AI at the *BRCA1 *locus is specifically associated with *BRCA1 *methylation. The genetic alterations at the *BRCA1 *locus were further examined by FISH, which revealed chromosome 17 deletions and heterogeneity with respect to chromosomal abnormalities. These results imply that methylation of the *BRCA1 *gene is accompanied by genomic rearrangements at the *BRCA1 *locus, resulting in loss of genetic material containing non-methylated *BRCA1 *alleles and retention of methylated *BRCA1 *alleles. We also found a substantially elevated frequency of *TP53 *mutations in the subset of *BRCA1 *methylated tumours, which has not been reported previously, suggesting that *BRCA1 *methylation might lead to alterations in the same molecular pathways as those known to be commonly altered in familial *BRCA1 *tumours. Collectively, these results implicate epigenetic silencing of the *BRCA1 *gene in sporadic breast tumorigenesis.

Medullary histological type was not found in the *BRCA1 *methylated tumours. However, we observed ER negativity to be significantly associated with *BRCA1 *methylation. We also found a substantial proportion of the *BRCA1 *methylated tumours to be of grade 3 and an indication for an association between *BRCA1 *methylation and early age of onset. Thus, our results indicate phenotypic similarities between *BRCA1 *methylated and familial *BRCA1 *breast tumours.

## Abbreviations

AI = allelic imbalance; CI = confidence interval; ER = estrogen receptor; FISH = fluorescence *in situ *hybridisation; OR = odds ratio; PR = progesterone receptor.

## Competing interests

The authors declare that they have no competing interests.

## Authors' contributions

VB and OAS contributed equally to this work, performing a substantial part of the analysis, participation in design and contribution to the writing of the manuscript. SKB contributed to FISH analysis, HH to *TP53* analysis and JGJ to supervision and analysis of pathological data and immunostaining. JEE conceived of the study, was in charge of its design and coordination and the writing of the manuscript. All authors read and approved the final manuscript.

## Supplementary Material

Additional file 1 An Excel file containing results of the FISH analysis along with DNA index and proportion of aneuploid cells. Click here for file

Additional file 2 An Excel file containing results for each of the microsattelite markers analysed for AI. Click here for file

## Figures and Tables

**Table 1:** Association between *BRCA1 *methylation and AI at the *BRCA *loci and other tumor features

Primary sporadic breast tumours	*BRCA1 *promoter methylation		P value
	Yes (*n *= 13)	No (*n *= 130)	

			
AI at the *BRCA1 *locus			
Yes	8 (62%)	41 (35%)	
No	5 (38%)	77 (65%)	0.0731
ND/NA		12	
			
AI at the *BRCA2 *locus			
Yes	5 (38%)	37 (30%)	
No	8 (62%)	85 (70%)	0.5420
ND/NA		8	
			
*TP53 *mutations			
Mutated *TP53 *gene	5 (38%)	22 (17%)	
Wild type	8 (62%)	106 (83%)	0.1299
ND/NA		2	
			
Estrogen receptor			
Negative	7 (54%)	33 (25%)	
Positive	6 (46%)	97 (75%)	0.0475
ND/NA			
			
Progesterone receptor			
Negative	6 (50%)	57 (44%)	
Positive	6 (50%)	72 (56%)	0.7699
ND/NA	1	1	
			
Age at diagnosis			
≤ 55 years	9 (69%)	57(44%)	
>55 years	4 (31%)	73 (56%)	0.0898

AI, allelic imbalance; ND/NA, not determined/not available.

**Table 2:** Phenotypic features of the *BRCA1 *methylated tumors

Tumour number	AI at *BRCA1*	AI at *BRCA2*	*TP53 *mutation	ER status	PR status	BRCA1 protein expression^a^	DNA index	Interpretation of FISH
1	+	-	-	+	+	1	NA	Deletion of the *BRCA1 *region
2	+	-	-	-	-	1	1.68	Deletion of the *BRCA1 *region
3	+	+	+	-	-	1	1.12; 1.99	Chromosome 17 deletion
4	+	+	+	-	-	1	1.54	Chromosome 17 deletion
5	+	+	+	+	+	1	1.22; 2.18	Chromosome 17 deletion
6	-	-	-	+	+	1	NA	Chromosome 17 deletion
7	+	-	+	-	-	1	3.32	No detectable *BRCA1 *deletion
8	-	-	-	+	+	1	1.11; 1.80	No detectable *BRCA1 *deletion
9	+	+	+	-	-	1	1.00	No detectable *BRCA1 *deletion
10	-	-	-	+	+	2	1.69	No detectable *BRCA1 *deletion
11	-	-	-	-	-	3	1.00	No detectable *BRCA1 *deletion
12	-	-	-	+	+	3	2.09	No detectable *BRCA1 *deletion
13	+	+	-	-	NA	3	NA	No detectable *BRCA1 *deletion

^a^BRCA1 protein expression in class 1 represents markedly reduced or absent expression; expression levels in class 2 and class 3 represent reduced and wild-type expression, respectively. AI, allelic imbalance; ER, estrogen receptor; NA, not available; PR, progesterone receptor.
